# Synergistic Antibacterial Action of Norfloxacin-Encapsulated G4 Hydrogels: The Role of Boronic Acid and Cyclodextrin

**DOI:** 10.3390/gels11010035

**Published:** 2025-01-04

**Authors:** Monica-Cornelia Sardaru, Irina Rosca, Simona Morariu, Elena-Laura Ursu, Alexandru Rotaru

**Affiliations:** 1 The Research Institute of the University of Bucharest (ICUB), 90 Sos. Panduri, 050663 Bucharest, Romania; sardaru.monica@icmpp.ro; 2 “Petru Poni” Institute of Macromolecular Chemistry, Romanian Academy, Centre of Advanced Research in Bionanoconjugates and Biopolymers, Grigore Ghica Voda Alley 41 A, 700487 Iasi, Romania; rosca.irina@icmpp.ro (I.R.); ursu.laura@icmpp.ro (E.-L.U.); 3 “Petru Poni” Institute of Macromolecular Chemistry, Romanian Academy, Natural Polymers, Bioactive and Biocompatible Materials, Grigore Ghica Voda Alley 41 A, 700487 Iasi, Romania; smorariu@icmpp.ro

**Keywords:** supramolecular hydrogel, guanosine quartet, *β*-cyclodextrin, *Staphylococcus aureus*, Norfloxacin

## Abstract

In this present study, we developed and characterized a series of supramolecular G4 hydrogels by integrating *β*-cyclodextrin (*β*-CD) and boronic acid linkers into a supramolecular matrix to enhance antibacterial activity against *Staphylococcus aureus* (*S. aureus*). We systematically investigated how varying the number of free boronic acid moieties (ranging from two to six), along with guanosine and β-CD content, influences both the structural integrity and antimicrobial efficacy of these materials. Comprehensive characterization using FTIR, circular dichroism, X-ray diffraction, SEM, AFM, and rheological measurements confirmed successful synthesis and revealed that higher boronic acid content correlated with a stronger, more organized network. The most effective hydrogel displayed an inhibition zone of 25 mm in disk diffusion assays, and was further explored as a drug delivery platform, with the aim to exploit the capacity of the free *β*-CD cavity of the hydrogels to incorporate hydrophobic drugs. Norfloxacin (Nfx), a poorly water-soluble antibiotic, was successfully encapsulated within the hydrogel matrix through the inclusion of complex formation with *β*-CD, improving its solubility and enabling sustained, targeted release. The Nfx-loaded hydrogel expanded the inhibition zone to 49 mm and completely eradicated *S. aureus* cells within 24 h, outperforming both the unloaded hydrogel and free Nfx. These results highlight the synergistic effect of boronic acid moieties and controlled drug release, underlining the potential of these hydrogels as versatile platforms for localized antimicrobial therapy, such as in wound dressings or implant coatings. Nevertheless, further in vivo studies and long-term stability assessments are needed to fully establish clinical relevance, safety, and scalability before these systems can be translated into routine healthcare applications.

## 1. Introduction

*Staphylococcus aureus* (*S. aureus*) is a highly adaptable and aggressive pathogen responsible for various infections in several environments, including dermal infections or critical diseases such as sepsis. It frequently colonizes human skin and nasal cavities, complicating eradication efforts and heightening the risk of invasive infections [1,2]. Antimicrobial resistance in *S. aureus* represents a significant threat to public health, as the emergence of multidrug-resistant strains limits treatment options, complicates infection management, and elevates the risk of severe clinical consequences [3,4,5,6].

Resistance to antibiotics can arise through several mechanisms [7], which can ultimately result in the reduced efficacy or complete loss of the medicine’s effectiveness [8,9]. Beyond microbial resistance, the application of antibiotics is constrained by other challenges, including complex synthesis processes, low aqueous solubility, limited or broad-spectrum activity, and reduced biocompatibility. Significant efforts are being dedicated to addressing these challenges and improving the efficacy of antibiotics. A series of innovative strategies was proposed to address the limitations of traditional antibiotics, focusing on the development of advanced systems [10], including metallic materials [11,12,13,14], carbon-based materials [15,16,17], and hydrogels with antimicrobial properties [18,19,20,21]. Hydrogels designed for biomedical applications are three-dimensional networks with hydrophilic structures, formed through physical interactions or covalent bonds, and are capable of absorbing and retaining significant amounts of water [22,23]. These materials exhibit unique properties like biocompatibility [21,24], tunable porosity [25,26], self-healing [27,28], responsiveness to stimuli [29,30,31,32], and controlled drug release [31,33], making them particularly promising candidates for next-generation antimicrobial platforms.

The diverse applications and versatility of hydrogels are largely influenced by the functional components within their structure. Cyclodextrins (CDs), which are non-toxic macromolecules capable of forming inclusion complexes with a variety of hydrophobic molecules [34], serve as crucial functional units that enhance the performance of hydrogels. Hydrogels containing CDs are particularly effective for the controlled release of bioactive molecules [35], making them highly suitable for wound dressing applications [36,37]. These materials combine the inherent benefits of hydrogels with the unique ability of CDs to encapsulate and release therapeutic agents.

Another functional component that enhances the antimicrobial properties of hydrogels is boronic acid or boronic acid groups. Boronic acids are effective antibacterial agents due to their ability to inhibit bacterial enzymes, such as serine *β*-lactamases, restoring the efficacy of *β*-lactam antibiotics [38,39,40]. They can chelate with diols on bacterial cell surface glycans, disrupting membrane integrity and metabolic processes. Additionally, boronic groups exhibit enhanced antibacterial activity when combined with other agents, such as in polymers or hydrogels, enabling controlled release and targeted binding. Their ability to disrupt and penetrate biofilms further highlights their potential [41,42].

Although numerous hydrogel systems incorporate CDs to enhance stability and potentially encapsulate therapeutic agents for various biomedical applications, such as cancer treatment, bone or cartilage repair, and myocardial infarction, many reported designs predominantly employ CDs as cross-linking agents [35,43]. Consequently, the amount of free or excess CDs available for forming inclusion complexes with hydrophobic drugs (e.g., dexamethasone or ellagic acid) is often minimal [35]. This limited presence of non-cross-linked CDs can restrict overall drug-loading capacity, particularly for lipophilic molecules, thereby narrowing effective therapeutic delivery. We have recently reported the development of an injectable supramolecular hydrogel containing *β*-CD cross-linked through guanosine quartets (G4), which exhibits exceptional and selective antimicrobial properties [44]. This advanced synthetic approach facilitates the precise and controllable covalent integration of *β*-CD units within the supramolecular matrix, enabling custom modulation of its physical properties. On the other hand, the reported system design further allows for modifications that introduce free boronic acid moieties, which may act as antimicrobial potentiators, enhancing the efficacy of the entire system. Herein, we present a series of five G4 hydrogel systems, designed to incorporate key structural components such as CDs and free boronic linker units with the aim of creating an efficient targeted antimicrobial drug delivery platform against *S. aureus* and to evaluate their capacity to enhance the solubility and controlled release of antibiotics. The proposed design offers the CD component exclusively to drug-loading thereby optimizing the solubility and controlled release of poorly water-soluble therapeutic agents. The most promising hydrogel candidate was subsequently loaded with Norfloxacin, a model broad-spectrum antibiotic with limited water solubility, known for the formation of “host-guest” inclusion complexes with CDs [45]. By exploiting the unique functional properties of cyclodextrin for the solubilization and controlled release of poorly soluble active compounds, alongside boronic acid groups for targeted antibacterial activity, these hydrogels present a versatile and potent platform for combating bacterial infections and thus advancing therapeutic innovation.

The developed antimicrobial hydrogel platforms were extensively characterized, and the impact of varying numbers of boronic acid moieties linked to cyclodextrin on *S. aureus* efficacy was systematically investigated. The drug-loaded hydrogel system was further evaluated for its ability to potentiate antibacterial activity, demonstrating the synergistic potential of the integrated design for enhanced therapeutic performance.

## 2. Results and Discussion

### 2.1. Synthesis of BdCD-G4 Hydrogels

**Scheme 1:**
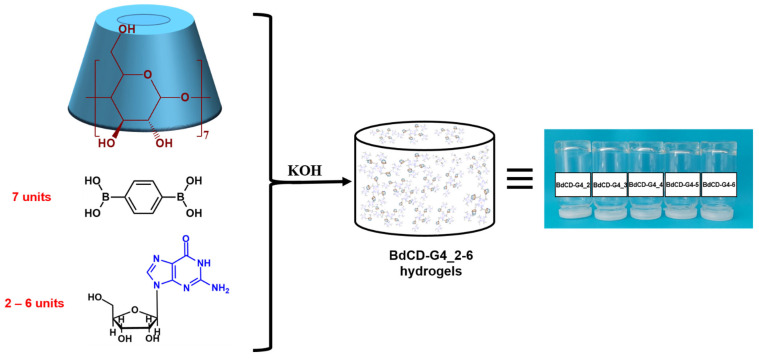
Schematic representation of BdCD-G4_2-6 hydrogel preparation; images of resulted self-standing transparent hydrogels.

Five supramolecular hydrogels (BdCD-G4_2-6) containing *β*-CD and variable amounts of boronic moieties were synthesized through a two-step procedure (Scheme 1, Table S1), using previously reported protocols [15,44,46]. In the initial step, *β*-CD was reacted with seven equivalents of benzene-1,4-diboronic acid in the presence of KOH, facilitating the covalent attachment of boronic acid linkers to the secondary hydroxyl groups of *β*-CD [47]. Subsequently, guanosine was added in equivalent amounts to promote the reticulation of the hydrogel system, driven by the self-assembly of guanosine into G4 structures in the presence of K^+^. Adding only one equivalent of guanosine to the system resulted in a weak and cloudy hydrogel, likely due to the insufficiency of a single guanosine unit attached to *β*-CD to effectively support the network structure. In contrast, the addition of two to six equivalents of guanosine produced transparent and robust hydrogels, designated as BdCD-G4_2, BdCD-G4_3, BdCD-G4_4, BdCD-G4_5, and BdCD-G4_6. The numerical indices correspond to the number of guanosine moieties linked to *β*-CD, indicating the degree of network integration and structural stability achieved.

The synthesis of BdCD-G4_2-6 hydrogels was reproducible, and the resulting hydrogels remained stable for several days at room temperature without changes in appearance. However, to advance towards industrial development and eventual clinical application, several key aspects must be addressed, including the scalability of the synthesis, as well as comprehensive long-term stability studies to ensure that these hydrogels maintain their structural integrity and functional characteristics under various storage and handling conditions.

### 2.2. Characterization of BdCD-G4 Hydrogels

#### 2.2.1. FTIR

**Figure 1:**
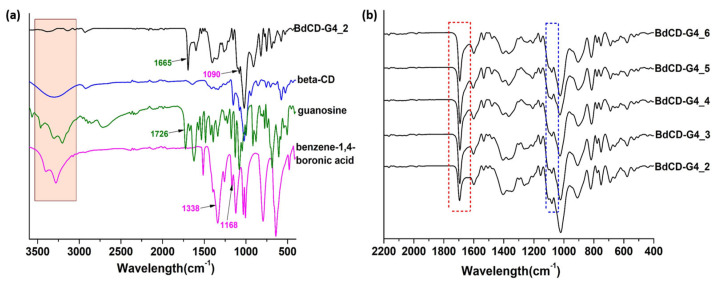
ATR-FTIR spectra for: (**a**) BdCD-G4_2 xerogel, and its precursors (*β*-CD, benzene-1,4-diboronic acid, and guanosine); (**b**) xerogels BdCD-G4_2-6.

A comprehensive characterization was carried out in order to obtain a better knowledge of the internal architecture and structural characteristics of the synthesized hydrogels. Figure 1a,b presents the ATR-FTIR spectra of the lyophilized hydrogels and each of their components to highlight particular synthesis-related changes.

In unmodified β-CD (Figure 1a), the O-H stretching vibrations are represented by the broad band observed in the range of 3500–3000 cm^−1^. Typically, the modification of secondary hydroxyl groups within the hydrogel structure results in a decreased intensity of this band, reflecting a reduced number of free hydroxyl groups. For benzene-1,4-diboronic acid, the FTIR spectrum showed two characteristic vibrations associated with the boronic group: the δB-OH vibration at 1168 cm^−1^ and the υB-O vibration at 1338 cm⁻^1^. In contrast, the FTIR spectra of the BdCD-G4-2 xerogel displayed a new υB-OC vibration at 1090 cm^−1^, replacing the δB-OH vibration at 1168 cm^−1^. This shift provides clear evidence of boronate ester formation, confirming the successful chemical integration of boronic groups into the hydrogel structure [48,49,50]. FTIR analysis also revealed the amide vibration of free guanosine molecules at 1726 cm^−1^, whereas in the xerogels, this vibration shifted to 1665 cm^−1^ due to the formation of G4. The hydrogel network’s guanosine molecules’ hydrogen bonding interactions are shown by this 61 cm^−1^ shift to a lower frequency [48,51]. The ATR-FTIR spectra of the BdCD-G4_2-6 xerogels (Figure 1b) exhibit no notable differences in their distinctive absorption bands, indicating a uniform structural composition among the samples, the principal bands associated with characteristic molecular vibrations, the υC=O stretching vibration of the amide bond at ≈ 1665 cm^−1^, and the υB-O vibration at ≈1090 cm^−1^.

#### 2.2.2. Circular Dichroism Investigations

To reveal the internal structure of BdCD-G4_2-6 hydrogels, we analyzed and compared their circular dichroism (CD) spectra. Thus, the samples from the settled reaction mixture were measured at 25 °C across a wavelength range of 220–340 nm (Figure S1). The CD spectra of all BdCD-G4_2-6 hydrogels exhibited two distinct features: a negative band near 260 nm, together with a positive band around 300 nm. The negative peak is indicative of guanosine analog assemblies, representing the formation of characteristic G4 structural units [46,52]. In contrast, the positive peak at 300 nm reflects *β*-CD stacking mediated by these G4 units, which drives the assembly of cyclodextrin–guanosine fibrils or nanowires [15,44].

#### 2.2.3. X-Ray Diffraction Analysis Investigations

To achieve an in-depth comprehension of the BdCD-G4_2-6 assembly pathway, we conducted a detailed structural analysis using X-ray diffraction (XRD) in our research. Two diffraction peaks were visible in the xerogels diffractograms (Figure S2, Table S2), indicating a supramolecular layered architecture. The PXRD patterns of the BdCD-G4_2-6 xerogels displayed unique patterns: a large diffraction peak with a center at 2θ ≈ 18.8° and a layer spacing of d ≈ 4.7 Å, which is indicative of channel-like assemblies that result from the orderly alignment and stacking of *β*-CD molecules [44,53]. The π–π stacking distance within G-quartet arrangements was also indicated by the presence of a second peak at approx. 26.5°, which corresponded to an intramolecular d-spacing of 3.4 Å [44,46]. The intensity of this peak exhibited a proportional increase with the number of guanosine units present in the investigated system, highlighting its dependency on G4 units.

#### 2.2.4. Scanning Electron Microscopy

**Figure 2:**
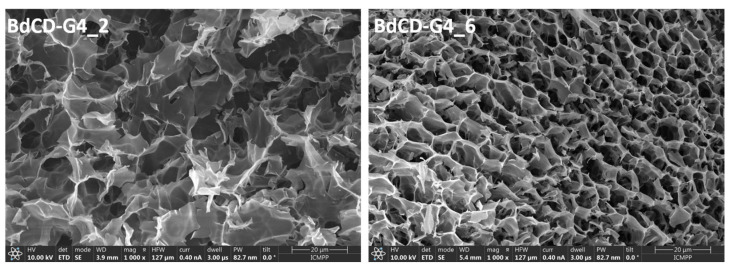
Representative SEM images for BdCD-G4_2 (**left**) and BdCD-G4_6 (**right**) xerogels (scale bar—20 µm).

The morphology at the microscopic level of the BdCD-G4_2-6 hydrogels was studied using scanning electron microscopy (SEM) on lyophilized hydrogel samples (Figure 2 and Figure S3). The SEM images reveal that the xerogel morphology and hydrogel network porosity were strongly influenced by the guanosine content in each sample. A lower guanosine content, as in case of the BdCD_G4_2 sample, led to the formation of an irregular network with undefined pores (Figure 2, left). In contrast, the BdCD-G4_6 hydrogel, which contains the maximum amount of grafted guanosine units, exhibited an ordered structure with small, well-defined pores (Figure 2, right).

#### 2.2.5. Atomic Force Microscopy

**Figure 3:**
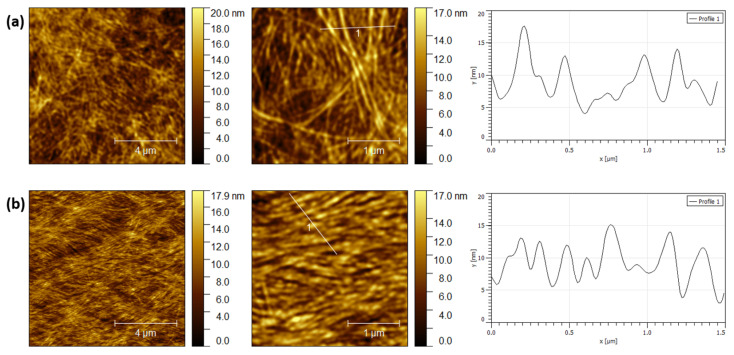
Examples of AFM images. (**a**) BdCD-G4_2: scale bar—4 µm (**left**), scale bar—1 µm (**middle**), Z-profiles along lines marked on images (**right**). (**b**) BdCD-G4_6: scale bar—4 µm (**left**), scale bar—1 µm (**middle**), Z-profiles along lines marked on images (**right**).

Morphological insights into the structural organization of hydrogels BdCD-G4_2-6 were revealed through AFM analysis (Figure 3 and Figures S4–S6).

The AFM images revealed, across all the investigated samples, distinct long fibrils up to several micrometers, flexible and interconnected with varying degrees of organization depending on the composition of each gel. For hydrogel BdCD-G4_2, the fibrillar structure appeared disorganized, with randomly distributed fibrils, indicating a less structured internal network (Figure 3a). However, as the number of guanosine units in the hydrogels increased, a clear transition toward well-organized fibrillar structures was observed, characterized by parallel alignments and increased density (Figure 3b). This progressive change highlights the significant influence of guanosine on the organization of the internal network. Also, Z-profile analysis showed an increase in fibril width as the number of guanosine units in the hydrogels increased, from a mean width of about 82 ± 11 nm in BdCD-G4_2 hydrogels (Figure 3a, right) to 88 ± 20 nm in BdCD-G4_6 hydrogels (Figure 3b, right) due to the lateral association of the G-quartet columnar stacks in the hydrogel network.

### 2.3. Rheological Investigations

**Figure 4:**
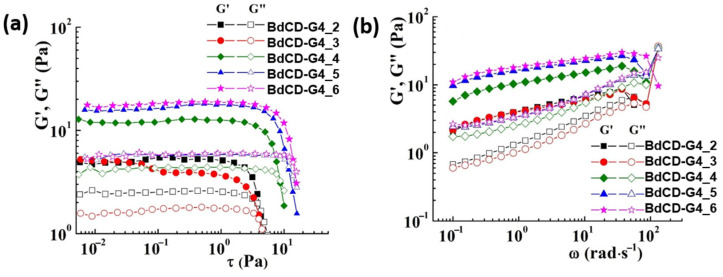
Amplitude and frequency sweep tests for BdCD-G4_2-6 samples at 25 °C: (**a**) G’ and G” as function of τ at 10 rad·s^–1^ and (**b**) variation in G’ and G” with ω at strain of 5%.

Amplitude sweep tests are essential for identifying the linear viscoelastic range (LVR) of materials, which is the region where both the storage modulus (G’) and loss modulus (G”) remain constant over the entire range of applied deformations. Figure 4 illustrates the results of amplitude sweep tests for the studied samples. In the low strain region, G’ and G” are independent of the stress, indicating the linear viscoelastic domain, and as the shear stress (τ) increases above the limit value of LVR, G’ and G” start to decrease, signifying the onset of non-linear viscoelastic behavior where the material structure begins to break down. All samples exhibit typical gel-like properties, with G’ being higher than G”. The LVR width and viscoelastic parameters show an expansion and an increase, respectively, by increasing the number of guanosine units, reflecting enhanced stability and a denser gel network from the BdCD-G4_2 sample to BdCD-G4_6. Thereby, the LVR limit rises from approximately 2 Pa for BdCD-G4_2 to 3.5 Pa for BdCD-G4_6 (Figure 4a).

In frequency sweep tests, the samples exhibit gel-like properties, as confirmed by linearity tests, with G’ and G” increasing with oscillation frequency (Figure 4b). The values of G’ and G”, at a strain of 5% and a frequency of 10 rad·s^–1^, range from 6.7 and 3.5 Pa to 24.4 and 7.3 Pa. The samples BdCD-G4_2 and BdCD-G4_3 have the lowest viscoelastic moduli, while BdCD-G4_6 has the highest values (Table S3). The increase in the number of linked guanosine molecules from two to six determines the increase in G’ and G”. Furthermore, the loss tangent (tan δ), which provides information about the viscoelastic behavior of materials, decreases from 0.52 for BdCD-G4_2 to 0.30 for BdCD-G4_6, indicating a slight structuring of the samples and improved elasticity. The close G’ and G” values observed between the samples BdCD-G4_2 and BdCD-G4_3, and the samples BdCD-G4_5 and BdCD-G4_6 indicate that structural changes, due to the increase in the number of linked guanosine molecules from two and five to three and six, respectively, do not significantly affect their viscoelastic behavior.

### 2.4. In Vitro Antibacterial Activity of BdCD-G4_2-6 Hydrogels

Based on previously reported hydrogels composed of cyclodextrin, 1,4-phenyldiboronic acid, and guanosine [15] that demonstrated acceptable cell viability (approximately 70% in MTS cell proliferation assays), we proceeded to evaluate the in vitro antibacterial activity of BdCD-G4_2-6 hydrogels to gain a comprehensive understanding of antimicrobial activity and assess the influence of free boronic groups. For comparison, we tested these formulations alongside the previously reported BdCD-G4_7 hydrogel [44], which contains no free boronic acid moieties. In the BdCD-G4_7 system, all seven boronic acid units fully reacted with cyclodextrin and guanosine, eliminating any free boronic groups in the hydrogel structure. The antibacterial activity of the sample resulting from disk diffusion assay are presented in Table 1 and Figure S7. All the tested hydrogels presented efficiency against *S. aureus* up to 25 mm in the case of the BdCD-G4_2 hydrogel. The analysis of the data reveals a strong correlation between the increase in free boronic groups and the enhancement of antibacterial activity against *S. aureus*. This fact highpoints the critical contribution of boronic functional groups in increasing the antimicrobial performance of the materials studied. The exceptional activity of the hydrogels and their mechanism of action can be explained by the established ability of boronic derivatives to selectively target the active sites of *S. aureus* caseinolytic protease P [42].

For potential applications of the BdCD-G4_2-7 hydrogels or their dried forms, sterilization presents a critical challenge, as conventional methods like heat sterilization or autoclaving may degrade the supramolecular architecture. Alternative sterilization techniques, such as UV [54,55] or gamma irradiation [56] and electron beam processing [57], should be explored to ensure that both the supramolecular network and the boronic ester bonds remain intact.

Given that the BdCD-G4_2 hydrogel demonstrated the strongest activity against *S. aureus* and contains the same amount of free *β*-CD molecules within its structure, further investigations focused on *β*-CD’s ability to encapsulate specific active compounds within its cavity. The primary objectives were to assess the enhancement in solubility of poorly water-soluble active principles and to evaluate the drug-loading capacity of the hydrogel system. Norfloxacin (Nfx) was selected as the drug model for encapsulation into the hydrogel. This compound was chosen as the active agent due to its dual properties: as a synthetic fluoroquinolone, it acts as a broad-spectrum antibacterial agent that inhibits bacterial DNA synthesis, targeting both Gram-negative and Gram-positive bacteria. Additionally, its limited water solubility and capacity to form inclusion complexes with *β*-CD make it an ideal candidate for testing the hydrogel’s performance as a solubility-enhancing and drug delivery platform [45,58].

### 2.5. Synthesis of BdCD-G4_2 Hydrogel Loading with Norfloxacin (BdCD-G4_2-Nfx)

**Scheme 2:**
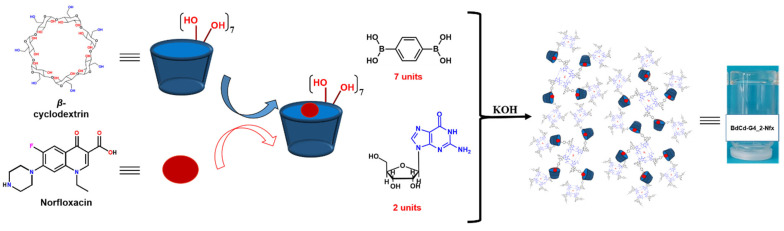
Schematic representation for formation of 3:1 inclusion complex between *β*-CD and Nfx, followed by synthesis of Nfx-loaded hydrogel (BdCD-G4_2-Nfx).

The synthesis of the BdCD-G4_2-Nfx formulation (Scheme 2) started with the preparation of an inclusion complex between *β*-CD and Nfx by combining water solutions of *β*-CD, KOH, and Norfloxacin in a 3:1 ratio.

After sonication and heating at 90 °C for 5 min, the solution became transparent, indicating the potential formation of the *β*-CD-Nfx inclusion complex. Next, the inclusion complex was incorporated into the hydrogel using the standard protocol for BdCD-G4_2 hydrogel preparation. The *β*-CD-Nfx complex was reacted with benzene-1,4-diboronic acid, followed by the addition of guanosine, resulting in the formation of a transparent hydrogel.

### 2.6. Characterization of BdCD-G4_2 Hydrogel Loading with Norfloxacin (BdCD-G4_2-Nfx)

#### 2.6.1. Structural Characterization

The formation of the inclusion complex between *β*-CD and Nfx was demonstrated through Raman spectroscopy [59]. Figure S8 shows the Raman spectra of Norfloxacin, cyclodextrins, and Norfloxacin inclusion complexes in the 1550–1700 cm⁻^1^ region. In the Raman spectrum of the guest–host complex, the peak at 1631 cm^−1^ (symmetric stretching of the C=O groups of the pyridone moiety and the stretching vibration of the C–C group of aromatic ring chain [60]) corresponding to free Norfloxacin is shifted by 4 cm^−1^, indicating the presence of guest–host interactions.

Additionally, a comparative analysis of the FTIR spectra (Figure S9) of the BdCD-G4_2 hydrogel and the Norfloxacin-loaded formulation BdCD-G4_2-Nfx revealed a shift in the υC=O vibration from 1616 cm^−1^ in the spectrum of free Norfloxacin to 1663 cm^−1^ in the formulation spectrum.

#### 2.6.2. X-Ray Diffraction

Next, the supramolecular arrangement of the structural units within the formulation was analyzed using X-ray diffraction. The X-ray diffractograms of the freeze-dried BdCD-G4_2 hydrogel and the BdCD-G4_2-Nfx formulation showed no visible differences (Figure S10). Both exhibited a diffraction peak at approximately 26º (the standard peak for the π-π distance between G-quartet stacks) and another broad diffraction peak at approximately 18° (a distinctive peak of channel-type assemblies formed by the stacking and alignment of *β*-CD molecules). Moreover, no crystalline peaks of Norfloxacin were observed in the formulation’s diffractogram, which indicates the successful encapsulation of the drug within the cyclodextrin cavity.

#### 2.6.3. Scanning Electron Microscopy (SEM)

To examine the impact of Nfx encapsulation on the morphological characteristics of the hydrogel, surface analyses were performed on the corresponding freeze-dried sample. SEM images (Figure S11) of the formulation revealed that drug encapsulation caused only minor alterations to the structure of the original hydrogel. The resulting structure appeared disorganized, similar to BdCD-G4_2, but with more defined pores and slightly thicker walls, indicating subtle changes in the hydrogel network.

### 2.7. In Vitro Antibacterial Activity of BdCD-G4_2-Nfx

The antibacterial activity of the samples (BdCD-G4_2, BdCD-G4-2_Nfx, and Norfloxacin), determined by the disk diffusion assay, is presented in Table 2 and Figure S12. Incorporating Norfloxacin into the hydrogels significantly enhanced their antimicrobial activity, with the inhibition zone increasing to 49 mm compared to reference BdCD-G4_2.

These results highlight the synergistic effect between the hydrogel matrix, particularly the boronic acid linkers, and cyclodextrin-encapsulated Nfx. Although free Nfx alone demonstrates antibacterial activity comparable to its CD inclusion complex [61,62], encapsulation within the hydrogel leverages the boronic acid moieties’ capacities to disrupt bacterial cell functions thereby amplifying the overall antimicrobial effect.

**Figure 5:**
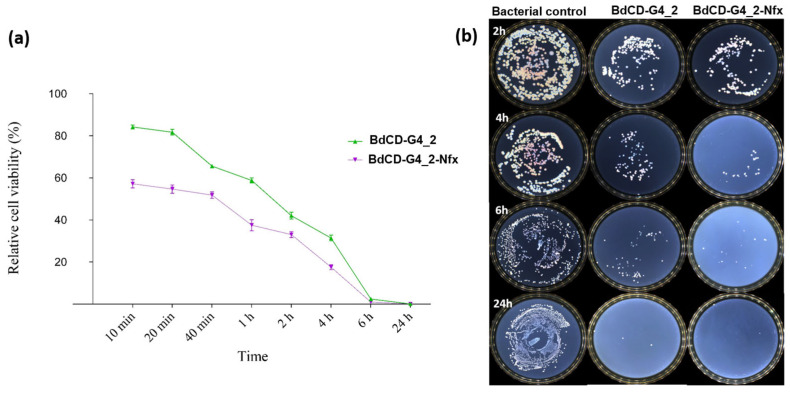
In vitro antibacterial activity. (**a**) Growth inhibition of bacterial colonies expressed as percent of viable bacteria vs. incubation time, and (**b**) *S. aureu* colonies after 2–24 h of incubation with xerogels.

Evaluating bacterial cell growth in the presence of the hydrogels revealed that the investigated hydrogels effectively inhibited *S. aureus*, with their efficiency increasing over time (Figure 5).

Sample BdCD-G4_2 exhibited superior performance, reducing bacterial activity to 65% within 40 min of incubation (Figure 5a). This effectiveness further intensified, achieving a remarkable 98% reduction in bacterial activity after 6 h. When loaded with Norfloxacin, the BdCD-G4_2-Nfx formulation exhibited even greater potency, eradicating over 99% of bacterial cells within the same period. Remarkably, after 24 h of incubation (Figure 5b), no viable bacterial cells were detected on the plates for any of the tested hydrogel formulations, highlighting their potent antibacterial properties.

## 3. Conclusions

In the current study, we designed and characterized a series of G4 hydrogels incorporating *β*-cyclodextrin and boronic acid linkers to achieve tunable antimicrobial activity against *Staphylococcus aureus*. Comprehensive analyses using FTIR, circular dichroism, X-ray diffraction, SEM, AFM, and rheological measurements confirmed successful synthesis and provided insights into their supramolecular organization. In vitro assays revealed that all formulations displayed antibacterial efficacy, with the BdCD-G4_2 hydrogel exhibiting the strongest performance by significantly reducing bacterial viability within hours. Furthermore, encapsulating poorly water-soluble Norfloxacin (Nfx) through *β*-cyclodextrin inclusion complexes not only enhanced the drug’s solubility, but also enabled sustained and targeted release. The BdCD-G4_2-Nfx hydrogel demonstrated complete eradication of *Staphylococcus aureus* within 24 h, outperforming both the unloaded hydrogel and free Nfx. These findings highlight the potential of this customizable hydrogel platform, offering a versatile and adaptive approach to antimicrobial therapy and targeted drug delivery.

## 4. Materials and Methods

### 4.1. Materials

The chemicals utilized in this study, such as guanosine, KOH, and Norfloxacin (98%), were purchased from Sigma (Schnelldorf, Germany). *β*-Cyclodextrin and benzene-1,4-diboronic acid were obtained from TCI (Tokyo, Japan). All reagents were used without any additional purification. Ultrapure water was consistently used in all experimental procedures and for the preparation of stock solutions.

### 4.2. Synthesis of BdCD-G4_2-6 Hydrogels

Five BdCD-G4_2-6 hydrogels were synthesized using an adapted protocol of a previously reported method [15,44,46]. The synthesis involved first mixing 0.025 mmols of *β*-CD (28.6 mg, 253 μL of a stock solution containing 0.565 g of *β*-CD in 5 mL of water), 0.176 mmols of benzene-1,4-diboronic acid (29.2 mg), and an equivalent volume of KOH stock solution (128.6 μL (12.6 mg) for BdCD-G4_2, 142.9 μL (14.0 mg) for BdCD-G4_3, 157.2 μL (15.3 mg) for BdCD-G4_4, 171.4 μL (16.3 mg) for BdCD-G4_5, or 185.7 μL (18.1 mg) for BdCD-G4_6) from a stock solution containing 0.488 g of KOH in 5 mL of water) in a glass vial with additional water to reach the volume of 2 mL. The mixture was sonicated for 2 min and then heated in an oil bath at 90 °C until the formation of a clear, transparent solution. Next, an equivalent amount of guanosine (14.3 mg for BdCD-G4_2, 21.6 mg for BdCD-G4_3, 28.6 mg for BdCD-G4_4, 35.8 mg for BdCD-G4_5, or 43 mg for BdCD-G4_6) was added, and the mixture was heated again at 90 °C until the complete solubilization of guanosine. Finally, the vial was allowed to cool to room temperature, resulting in the formation of a stable, transparent hydrogel with a total volume of 2 mL, as detailed in Table S1.

### 4.3. Synthesis of Hydrogel Loading with Norfloxacin (BdCD-G4_2-Nfx)

The synthesis of the BdCD-G4_2-Nfx formulation begins with the preparation of the inclusion complex between *β*-CD and Norfloxacin (Nfx). Thus, 0.025 mmols of *β*-CD (28.6 mg, 253 μL of a stock solution containing 0.565 g of *β*-CD in 5 mL of water), 0.013 mM of Norfloxacin (4 mg), and 0.22 mM of KOH (128.6 μL of a stock solution containing 0.488 g of KOH in 5 mL of water) were mixed in 1619 μL of water. The mixture was sonicated for 5 min and then heated in an oil bath at 90 °C until the cloudy solution became transparent. Next, the process continues as described in the hydrogel synthesis protocol. Specifically, 0.176 mmoli of benzene-1,4-diboronic acid (29.2 mg) and 0.05 mmoli of guanosine (14.3 mg) were added. When all components were fully dissolved, the vial was left to cool to room temperature, resulting in the formation of a transparent BdCD-G4_2-Nfx hydrogel.

### 4.4. Equipment and Methods

#### 4.4.1. Fourier Transform Infrared (FTIR) Spectroscopy

The FTIR spectra of BdCD-G4_2-6 xerogels and formulations were obtained using the ATR method on an INVENIO-R FTIR spectrometer (Bruker, Ettingen, Germany). The measurements included 90 scans at a resolution of 4, covering a spectral range of 4000–400 cm⁻^1^. The spectra were processed using OPUS 8.7.41 (Bruker, Ettingen, Germany) and OriginPro 8 software (Northampton, MA, USA).

#### 4.4.2. Circular Dichroism

Circular dichroism (CD) measurements were performed using a Chirascan plus instrument (Applied Photophysics Ltd., Leatherhead, Surrey, UK). The CD spectra were recorded in the range of 340 to 220 nm, with a data pitch of 0.5 nm at an acquisition speed of 200 nm/min. The experiment consisted of taking 100 µL of the reaction mixture sample and placing it into 1 mm lamellas. The spectra were processed using OriginPro 8 software (Northampton, MA, USA).

#### 4.4.3. Scanning Electron Microscopy (SEM)

The hydrogel structures were analyzed via scanning electron microscopy (SEM) using the FEI NanoSEM430 instrument (Hillsboro, OR, USA). Prior to measurement, the hydrogel samples were subjected to freeze drying.

#### 4.4.4. X–Ray Diffraction

X-ray diffraction analysis was conducted using a Rigaku SmartLab X-ray diffractometer (Rigaku Corporation, Tokyo, Japan) in an angular range of 5–60° (2θ), with a scanning step of 0.03° and a recording rate of 5°/min. OriginPro 8 program (Northampton, MA, USA) was utilized for the processing of the spectra.

#### 4.4.5. Atomic Force Investigation

AFM investigation was carried out using an Ntegra Spectra Atomic Force Microscope (NT-MDT Spectrum Instruments, Zelenograd, Moscow, Russia) in tapping mode under ambient conditions. The study utilized silicon cantilever tips with gold reflecting coating (NSG03, ScanSens, Bremen, Germany). Aliquots of 10 µL of the hydrogel solution were evenly applied to freshly cleaved mica substrates and air-dried at room temperature before imaging. AFM image analysis was performed using Gwyddion 2.59 software (Brno, Czech Republic) [63].

#### 4.4.6. Rheological Measurements

Rheological tests were performed at 25 °C on an MCR302 rheometer (Anton Paar, Graz, Austria) using plane–plane geometry with a diameter of 25 mm. A Peltier device ensures temperature control. Amplitude sweep tests were carried out in the shear stress range of 5 × 10^–3^–2 × 10 Pa (corresponding to a strain range of 5 × 10^–2^–10^3^%) at 10 rad·s^−1^ in order to determine the linear viscoelastic regime (LVR) of the investigated samples. Frequency sweep measurements were performed in the frequency (ω) range of 10^–1^–2 × 10^2^ rad·s^–1^ at a strain of 5% from the linear viscoelastic range.

#### 4.4.7. Raman Measurements

Raman spectra on lyophilized samples were acquired using an InVia Raman Microscope (Renishaw, Gloucestershire, UK) equipped with a He–Ne laser at 632.8 nm (17 mW) and a CCD detector coupled to a Leica DM 2500 M microscope. All measurements were performed in backscattering geometry using a 50× objective (NA = 0.75, N Plan EPI, Leica, Wetzlar, Germany). Three scans were accumulated for each spectrum with laser exposure for each scan of 10 sec to achieve an appropriate signal-to-noise ratio. Spectral manipulations were performed with the WiRE 3.2 software (Renishaw, Gloucestershire, UK).

#### 4.4.8. Antimicrobial Properties

The antibacterial activity of the hydrogels and controls was determined by disk diffusion assay [64,65] against a Gram-positive bacterial strain represented by *S. aureus* ATCC 25923. The microorganism was stored at -80 °C in 20% glycerol. The bacterial strain was refreshed in nutrient broth (NB) at 37 °C and microbial suspension was prepared with this culture in a sterile solution to obtain turbidity, optically comparable to that of 0.5 McFarland standards. Volumes of 0.1 mL from this inoculum were spread onto nutrient agar (NA) plates. Xerogel samples with a 10 mm diameter and 20 mg were added on the inoculated plates. To evaluate the antibacterial properties, growth inhibition was measured under standard conditions after 24 h of incubation at 37 °C.

Viable cell-counting method, which evaluates kinetics of bacterial cell growth [66], was also performed in order to quantify the antibacterial activity of the hydrogels against the reference bacterial strain. The samples were (20 mg) placed into solutions, which contained 0.5 mL of the bacterium suspensions and 4.5 mL 1X PBS solution, followed by incubation in a shaker at 37 °C up to 24 h. The following control experiment was conducted: 1 µL of the control sample and of the treated samples were removed at determined periods of incubation time (10 min, 20 min, 40 min, 1 h, 2 h, 3 h, 6 h, and 24 h) and spread on plate count agar (PCA) plates. The number of colonies was counted after 24 h of incubation at 37 °C.

All tests were carried out in triplicate to verify the results. After incubation, the plates were analyzed with SCAN1200^®^, version 8.6.10.0 (Interscience, Saint Nom la Bretêche, France), and the inhibition zones/number of colonies was expressed as the mean ± standard deviation (SD), performed with GraphPad Prism software version 7.00 for Windows (GraphPad Software, La Jolla, CA, USA, www.graphpad.com). The ratio of the inhibited growth of the bacteria (IRG) was calculated for the second experiment as follows:

(1) $$IRG\left(\%\right)=\frac{N_{a}-N_{b}}{N_{b}}\cdot 100$$

where *N_a_* and *N_b_* are the average values of the colonies of the control group and the experimental groups, respectively.

## Figures and Tables

**Table 1:** The antibacterial activity of the BdCD-G4_2-7 hydrogels against the reference strain.

Sample	BdCD-G4_2	BdCD-G4_3	BdCD-G4_4	BdCD-G4_5	BdCD-G4_6	BdCD-G4_7
Inhibition Zone (mm) *S. aureus*	24.80 ± 1.08	23.20 ± 1.65	21.20 ± 0.55	21.13 ± 0.92	19.03 ± 0.92	17.40 ± 0.17

**Table 2:** Antibacterial activity of BdCD-G4_2, BdCD-G4_2-Nfx, and Norfloxacin, determined by disk diffusion assay against reference strain.

Sample	Norfloxacin	BdCD-G4_2	BdCD-G4_2-Nfx
Inhibition Zone (mm) *S. aureus*	38.56 ± 0.05	24.80 ± 1.08	48.63 ± 0.58

## Data Availability

The original contributions presented in this study are included in the article/Supplementary Material. Further inquiries can be directed to the corresponding author.

## Supplementary Materials

The following supporting information can be downloaded at: https://www.mdpi.com/article/10.3390/gels11010035/s1. Table S1: Ratios of the precursors for the preparation of hydrogels BdCD-G4_2-6; Figure S1: CD spectra of BdCD-G4_2-6 hydrogels in the 220-340 nm range; Figure S2: X-Ray diffraction patterns of BdCD-G4_2-6 xerogels; Table S2: The diffraction peaks and the intermolecular d-spacing for hydrogels BdCD-G4_2-6; Figure S3: Examples of SEM images for BdCD-G4_3-5 xerogels (Scale bar—20 µm).; Figure S4: AFM images of BdCD-G4_3: scale bar—4 µm (left), scale bar—1 µm (middle), Z-profiles along the lines marked on the images (right); Figure S5: AFM images of BdCD-G4_4: scale bar—4 µm (left), scale bar—1 µm (middle), Z-profiles along the lines marked on the images (right); Figure S6: AFM images of BdCD-G4_5: scale bar—4 µm (left), scale bar—1 µm (middle), Z-profiles along the lines marked on the images (right); Table S3: Rheological parameters of the BdCD-G4_2-6 hydrogels; Figure S7: Antibacterial activity of BdCD-G4_2-7 hydrogels against *S. aureus* determined by disk diffusion assay; Figure S8: Raman spectra of β-CD, Norfloxacin (Nfx), and the Nfx: β-CD inclusion complex (β-CD+Nfx) in the 1550–1700 cm^−1^ wavenumber ranges; Figure S9: FTIR spectra of Norfloxacin (Nfx), BdCD-G4_2, and formulation BdCD-G4_2-Nfx; Figure S10: X-Ray diffraction patterns of freeze-dried Norfloxacin (Nfx), BdCD-G4_2, and BdCD-G4_2-Nfx formulation; Figure S11: SEM images of BdCD-G4_2-Nfx formulation, scale bar—10 µm (left) and scale bar—20 µm (right); Figure S12: Antibacterial activity against *S. aureus* determined by disk diffusion assay for: (a) Norfloxacin (Nfx); (b) BdCD-G4_2 hydrogel; (c) BdCD-G4_2-Nfx formulation.
