# Supplementary material for: Synergistic Antibacterial Action of Norfloxacin-Encapsulated G4 Hydrogels: The Role of Boronic Acid and Cyclodextrin
Source: Gels. 2025 Jan 4;11(1):35. doi: 10.3390/gels11010035 (PMC11764500; doi:10.3390/gels11010035)
Supplement: Supplementary file 1 [file gels-11-00035-s001.zip › gels-3376828-supplementary.pdf]

Synergistic Antibacterial Action of Norfloxacin-Encapsulated G4 Hydrogels: The Role of Boronic Acid and Cyclodextrin

*Monica-Cornelia Sardaru, Irina Rosca, Simona Morariu, Elena-Laura Ursu, and Alexandru Rotaru**

**Correspondence: rotaru.alexandru@icmpp.ro*

Content

1. The composition of hydrogels – **Table S1**
2. CD spectra of hydrogels – **Figure S1**
3. Powder X-ray diffractions – **Figure S2, Table S2**
4. SEM images of hydrogels – **Figure S3**
5. AFM images of hydrogels – **Figures S4-S6**
6. Rheological Investigations – **Table S3**
7. *In vitro* Antibacterial Activity of hydrogels – **Figure S7**
8. Raman Spectroscopy – **Figure S8**
9. FTIR spectrum of formulation – **Figure S9**
10. Powder X-ray diffractions of formulation – **Figure S10**
11. SEM images of formulation – **Figure S11**
12. *In vitro* Antibacterial Activity of formulation– **Figure S12**

Table S1. Ratios of the precursors for the preparation of hydrogels **BdCD-G4_2-6**

Sample	*β*-CD stock solution (μl)	1,4 - benzene diboronic acid (mg)	Guanosine (mg)	KOH stock solution (μl)	Water volume (μl)	Total volume of hydrogel (μl)
**BdCD-G4_2**	253	29.2	14.3	128.6	1618.4	2000
**BdCD-G4_3**	253	29.2	21.6	142.9	1604.1	2000
**BdCD-G4_4**	253	29.2	28.6	157.2	1589.8	2000
**BdCD-G4_5**	253	29.2	35.8	171.4	1575.6	2000
**BdCD-G4_6**	253	29.2	43.0	185.7	1561.3	2000

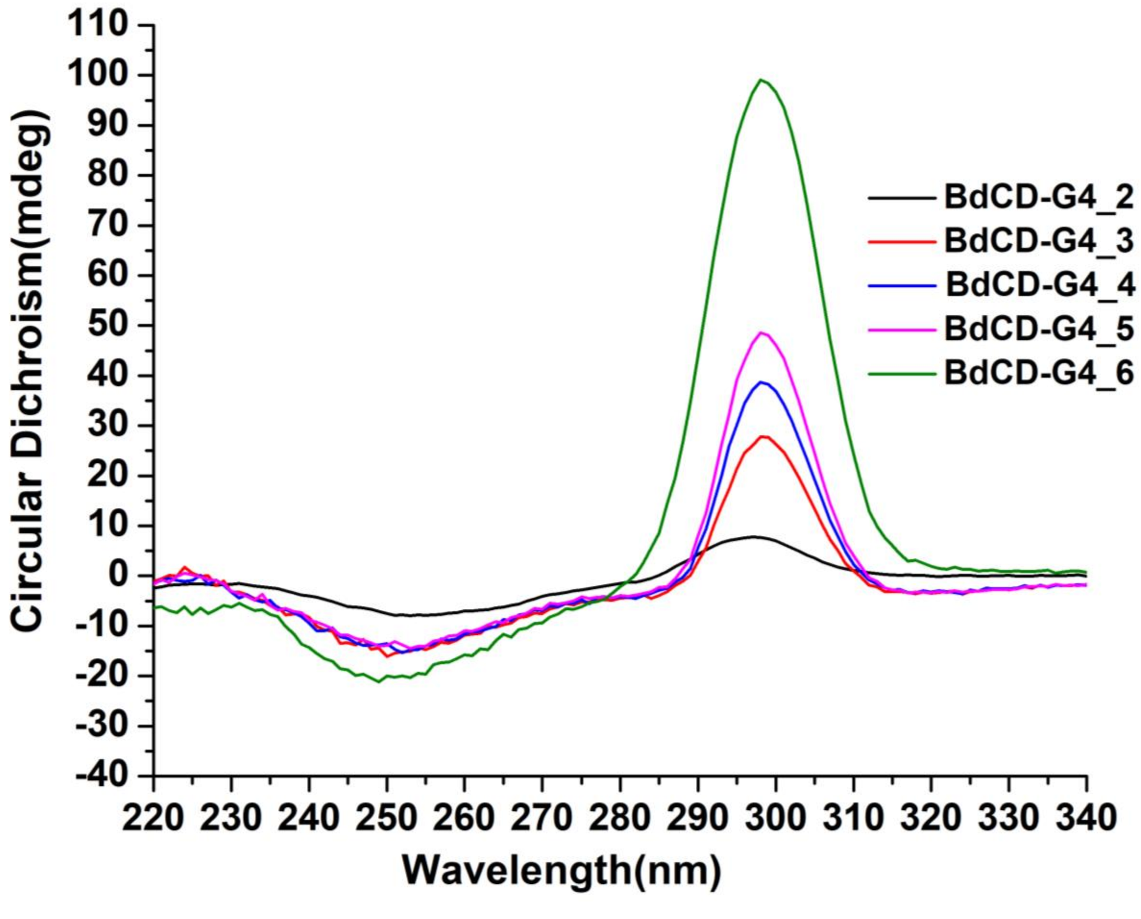

Figure S1. CD spectra of **BdCD-G4_2-6** hydrogels in the 220-340 nm range.

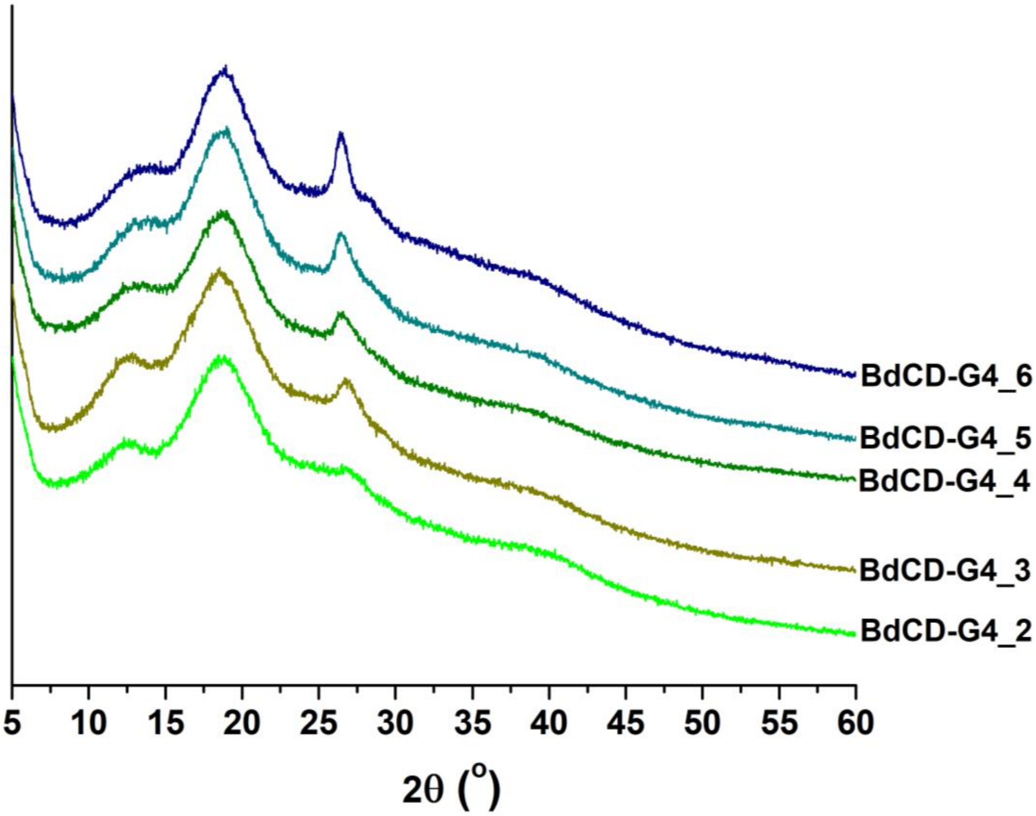

Figure S2. X-Ray diffraction patterns of **BdCD-G4_2-6** xerogels.

Table S2. The diffraction peaks and the intermolecular d-spacing for hydrogels **BdCD-G4_2-6**

**Sample**	***2Theta(˚)***	**d(Å)**	***2Theta(˚)***	**d(Å)**
**BdCD-G4_2**	18.8	4.7	26.7	3.3
**BdCD-G4_3**	18.6	4.8	26.8	3.3
**BdCD-G4_4**	18.6	4.8	26.5	3.4
**BdCD-G4_5**	18.6	4.8	26.5	3.4
**BdCD-G4_6**	18.7	4.7	26.4	3.4

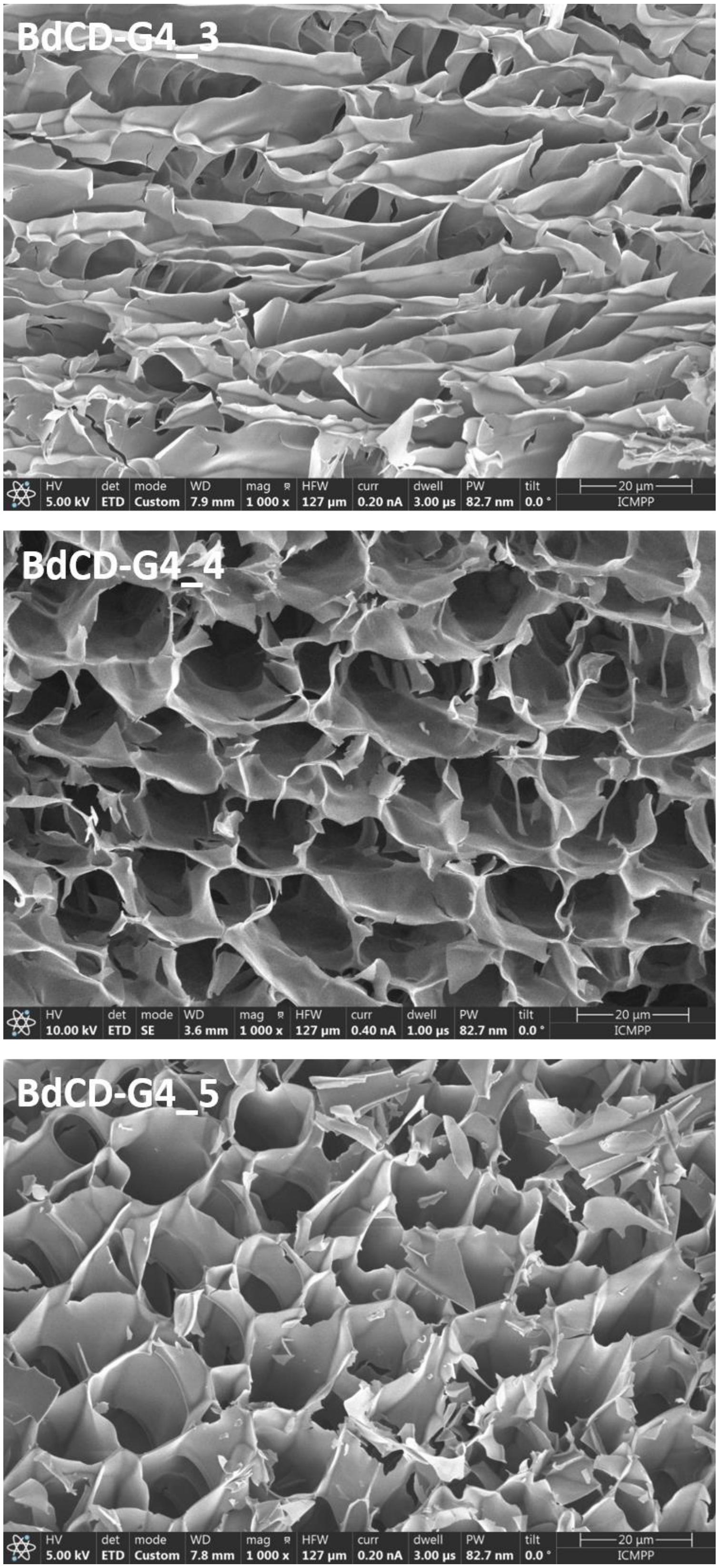

Figure S3. Examples of SEM images for **BdCD-G4_3-5** xerogels (Scale bar - 20 µm).

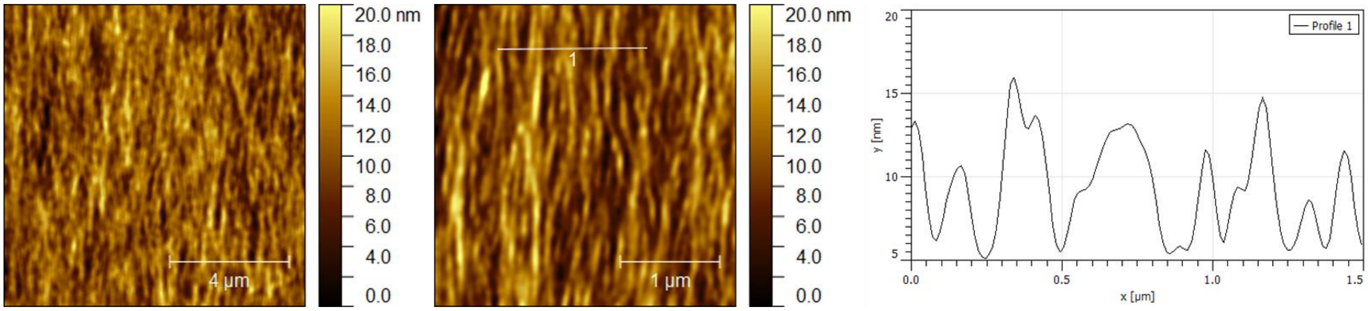

Figure S4. AFM images of **BdCD-G4_3**: scale bar – 4 µm (**left**), scale bar – 1 µm (**middle**), Z-profiles along the lines marked on the images (**right**).

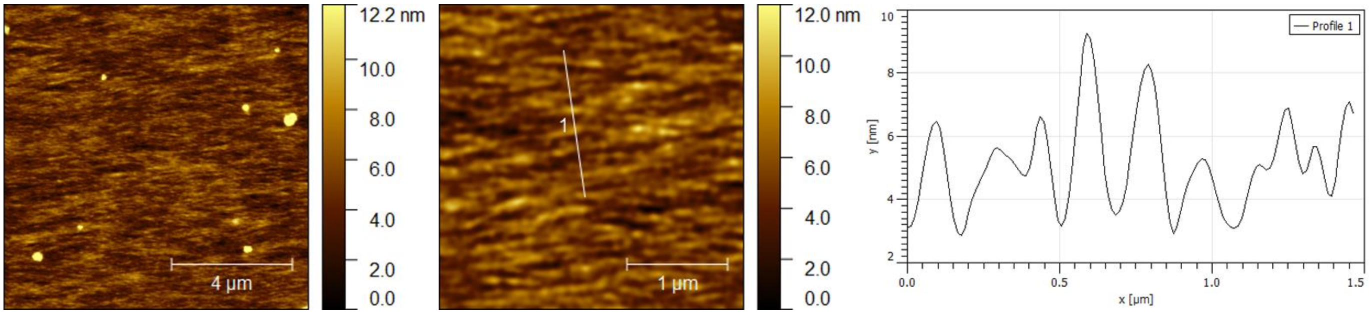

Figure S5. AFM images of **BdCD-G4_4**: scale bar – 4 µm (**left**), scale bar – 1 µm (**middle**), Z-profiles along the lines marked on the images (**right**).

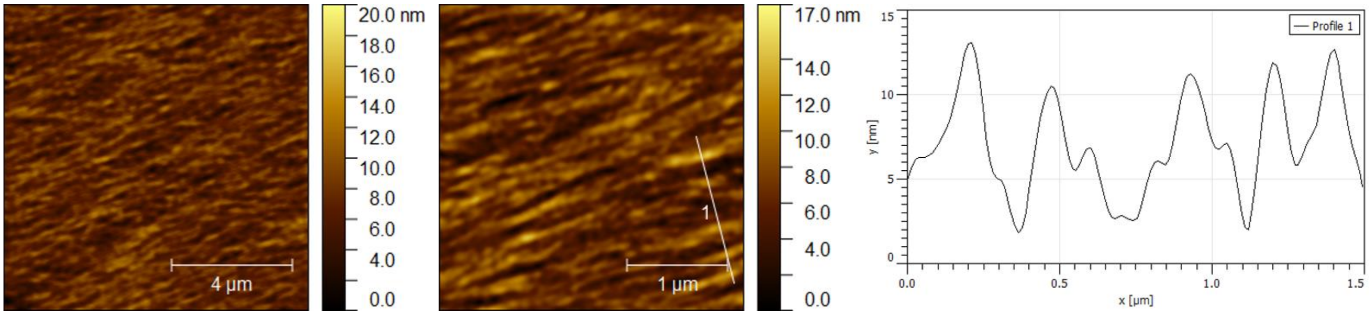

Figure S6. AFM images of **BdCD-G4_5**: scale bar – 4 µm (**left**), scale bar – 1 µm (**middle**), Z-profiles along the lines marked on the images (**right**).

Table S3. Rheological parameters of the **BdCD-G4_2-6** hydrogels

Sample	G'[a] (Pa)	G''[a] (Pa)	tan δ[a]	η_0[b] (Pa·s)	n[b]	η*[c] before applying high γ (Pa·s)	η*[c] recovery after 60 s (%)	η*[c] recovery after 600 s (%)
**BdCD-G4_2**	6.7	3.5	0.52	67.7±0.4	0.08±0.02	0.46	68.1	97.7
**BdCD-G4_3**	6.0	2.7	0.45	98.8±1.2	0.09±0.04	0.60	73.5	99.3
**BdCD-G4_4**	15.2	5.5	0.36	213.8±3.8	0.16±0.05	1.17	74.9	100
**BdCD-G4_5**	22.8	7.1	0.31	302.7±4.9	0.09±0.06	1.50	77.2	100
**BdCD-G4_6**	24.4	7.3	0.30	420.6±11.8	0.07±0.08	2.17	78.9	100

[a] values provided by the frequency sweep measurements at 5% and 10 rad·s^{-1}; tan δ was calculated as the G"/ G' ratio;

[b] determined by fitting the experimental data with eq. 2;

[c] the complex viscosity, η*, before applying a strain of 500%, and the percentage of η* regeneration after the stress removal.

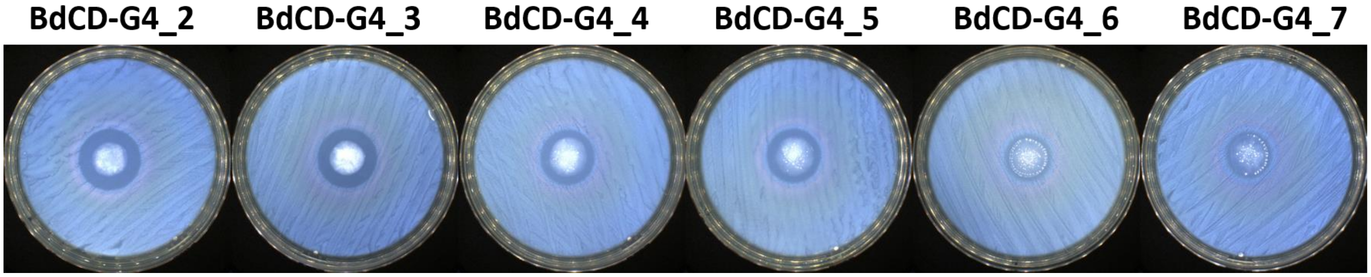

Figure S7. Antibacterial activity of **BdCD-G4_2-7** hydrogels against *S. aureus* determined by disk diffusion assay.

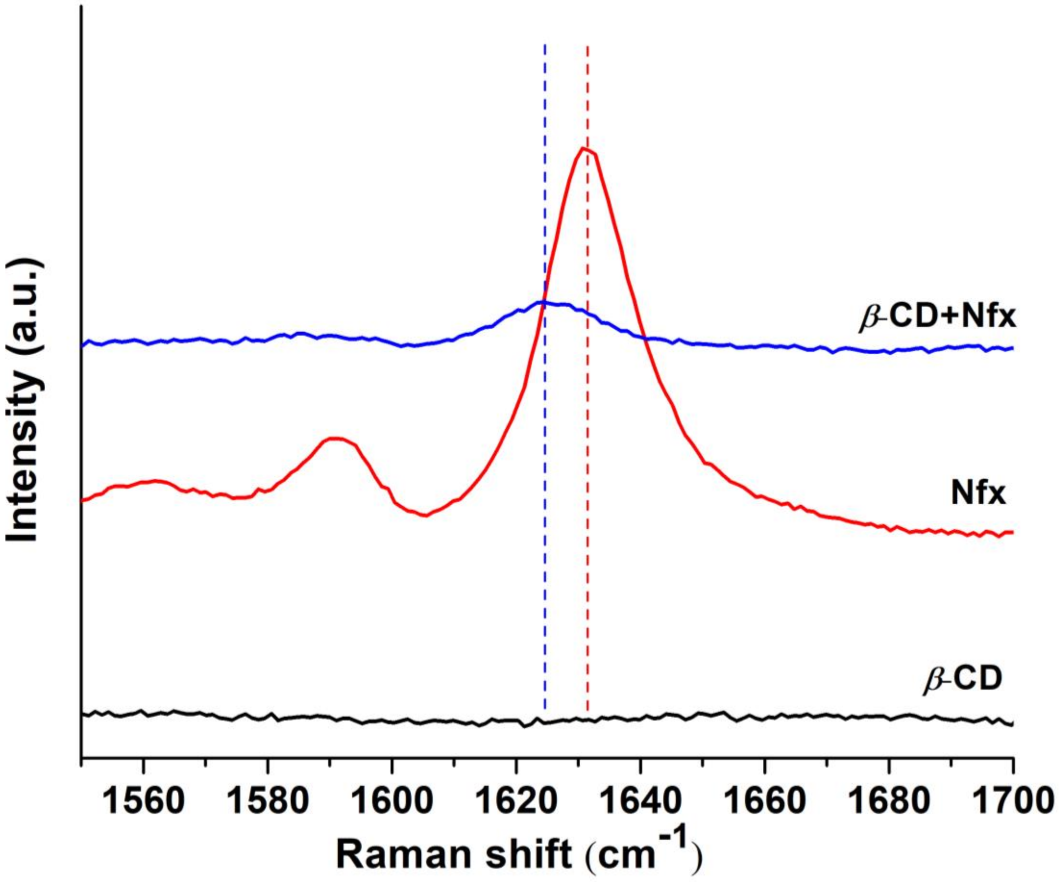

Figure S8. Raman spectra of ***β*-CD**, **Norfloxacin (Nfx)**, and the **Nfx : *β*-CD** inclusion complex (*β*-CD+Nfx) in the 1550 - 1700 cm^{-1} wavenumber ranges.

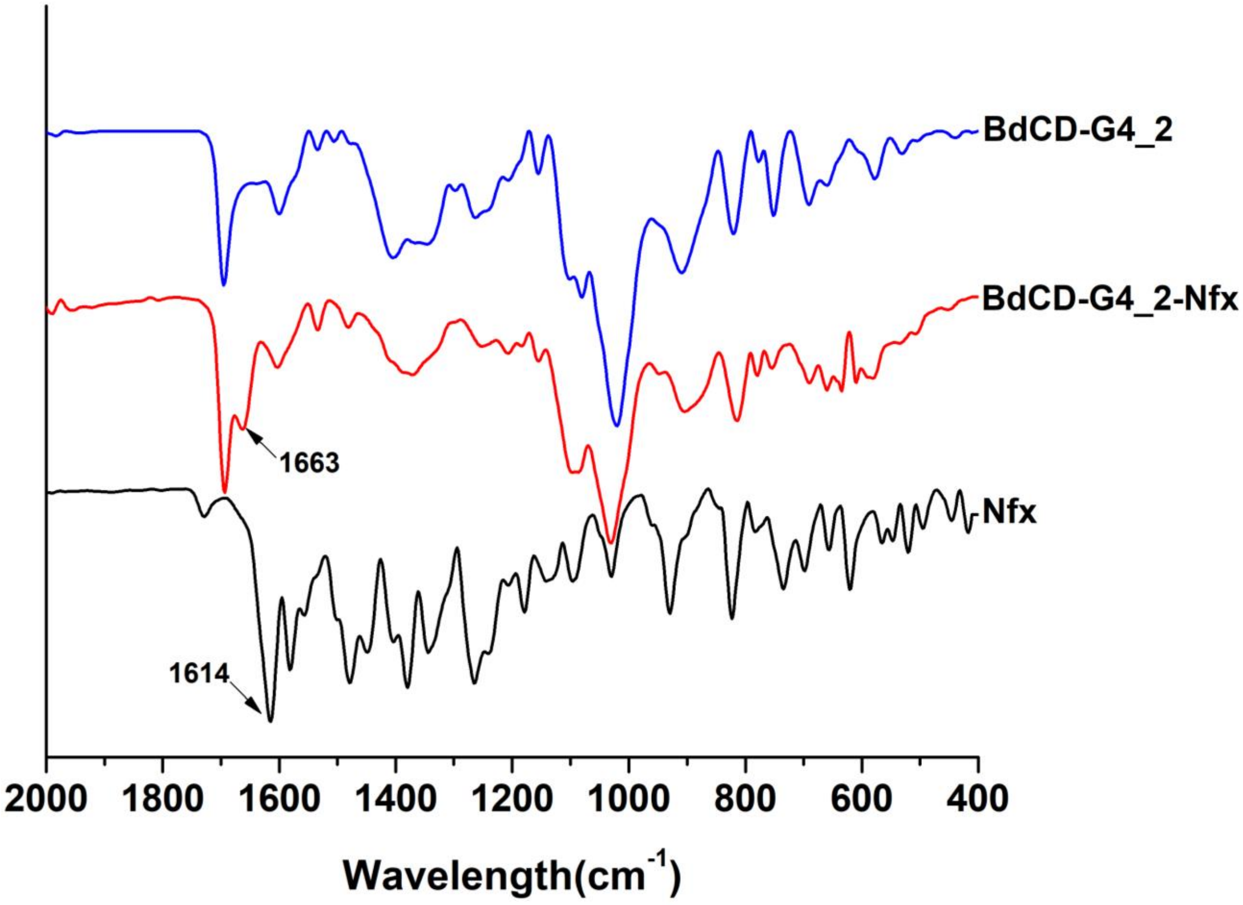

Figure S9. FTIR spectra of **Norfloxacin (Nfx)**, **BdCD-G4_2**, and formulation **BdCD-G4_2-Nfx**.

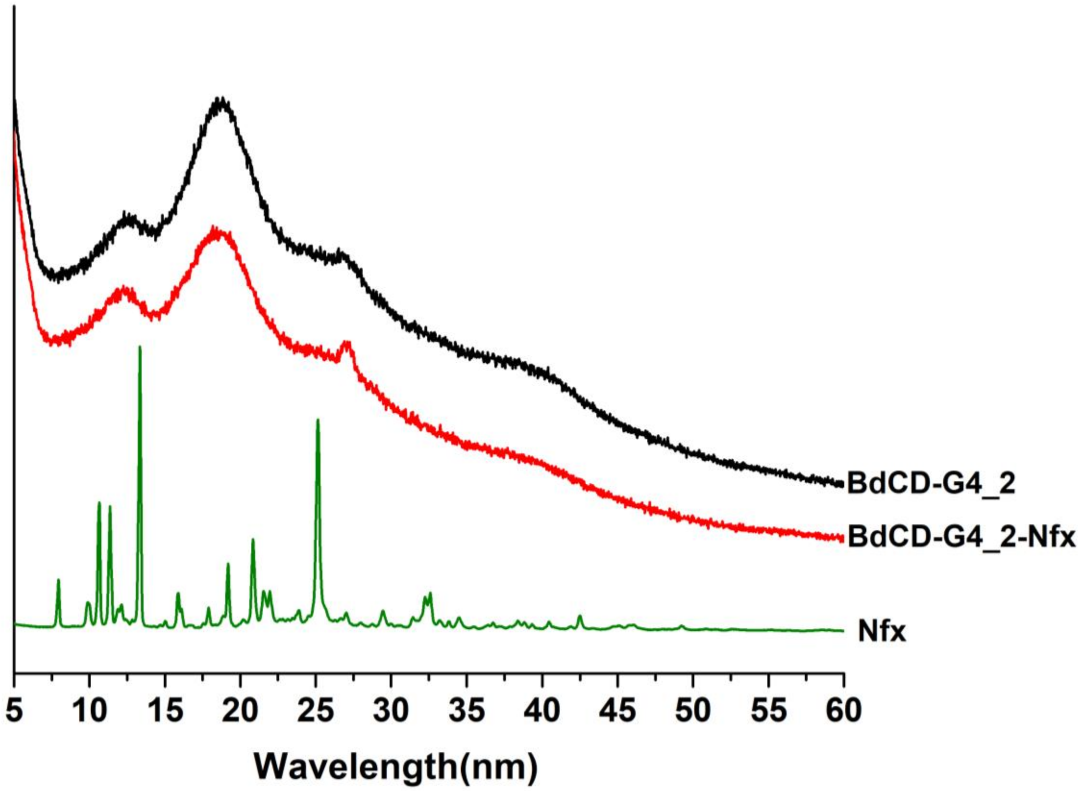

Figure S10. X-Ray diffraction patterns of freeze-dried **Norfloxacin (Nfx)**, **BdCD-G4_2**, and **BdCD-G4_2-Nfx** formulation.

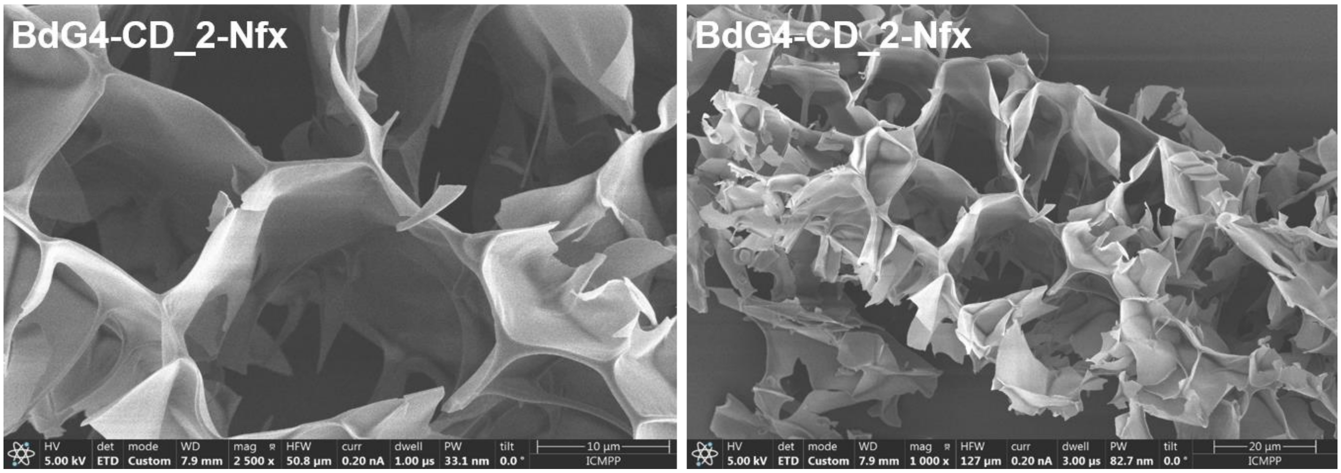

Figure S11. SEM images of **BdCD-G4_2-Nfx** formulation, scale - bar 10 µm (left) and scale - bar 20 µm (right).

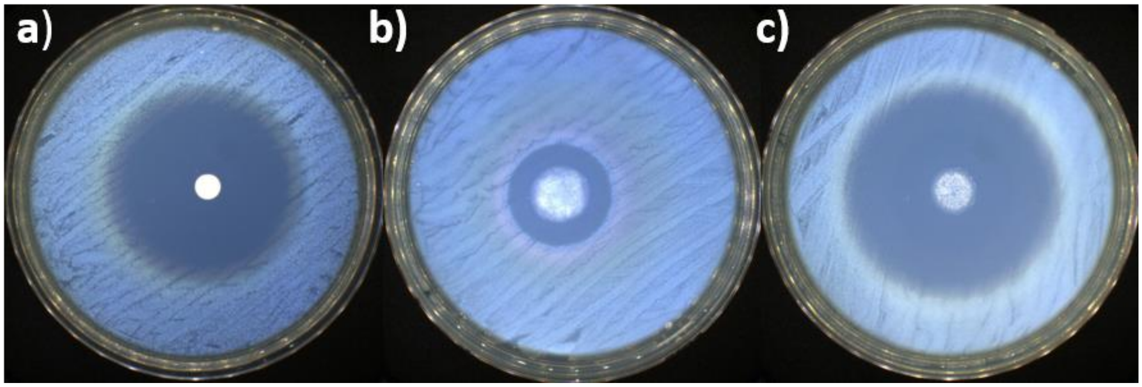

Figure S12. Antibacterial activity against *S. aureus* determined by disk diffusion assay for: **a) Norfloxacin (Nfx)**; **b) BdCD-G4_2** hydrogel; **c) BdCD-G4_2-Nfx** formulation.
